# Spatio-temporal parameters of ataxia gait dataset obtained with the Kinect

**DOI:** 10.1016/j.dib.2020.106307

**Published:** 2020-09-11

**Authors:** S. Summa, G. Tartarisco, M. Favetta, A. Buzachis, A. Romano, G.M. Bernava, G. Vasco, G. Pioggia, M. Petrarca, E. Castelli, E. Bertini, T. Schirinzi

**Affiliations:** 1 MARlab, Neuroscience and Neurorehabilitation Department, Bambino Gesù Children's Hospital – IRCCS, Rome, Italy; 2 National Research Council of Italy (CNR)–Institute for Biomedical Research and Innovation (IRIB), Messina Italy; 3 Department of Mathematics and Computer Science, University of Messina, Italy; 4 Unit of Neuromuscolar and Neurodegenerative Diseases, Department of Neurosciences, IRCCS Bambino Gesù Children's Hospital, Rome, Italy; 5 Department Systems Medicine, University of Roma Tor Vergata, Rome, Italy

**Keywords:** Gait analysis, Spatio-temporal parameters, Kinect, Ataxia

## Abstract

Ataxic syndromes include several rare, inherited and acquired conditions. One of the main issues is the absence of specific, and sensitive automatic evaluation tools and digital outcome measures to obtain a continuous monitoring of subjects' motor ability.

Gait evaluation was performed by Kinect v2 in a cohort of young participant affected by ataxia syndrome. The dataset is composed of the spatio-temporal parameters calculated by the skeleton acquired by the Kinect sensor, by the diagnosis of each participant, and by the total score of the clinical scale SARA. These parameters have been previously validated and corrected as requested by the Bland-Altman test.

## Specifications Table

**Table:** 

Subject	Biomedical Engineering
Specific subject area	Gait analysis is a discipline that systematically studies the human motion in terms of biomechanics.
Type of data	Table
How data were acquired	Instruments: Kinect v2, Tripod, Microsoft Kinect SDK.
Data format	Filtered Analyzed Corrected – following Bland-Altman test instructions.
Parameters for data collection	Participants’ gait was collected in a room of 120 m^2^ illuminated only with artificial lights in order to not interfere with the IF sensor.
Description of data collection	Data were acquired by a Kinect v2 placed on a tripod (tilt angle 0°), in front of the participant so that the frontal view was obtained and at a height of 1 m. In order to ensure that a minimum of one full gait cycle per leg was captured we placed the Kinect at a distance of 4.5 m from the starting line and we asked to the patient to walk barefoot at their self-selected speed until the stopping line that was placed 1.5 m from the Kinect, avoiding from the gait cycle analyzed the starting and ending acceleration and deceleration phases.
Data source location	Movement Analysis and Robotics Laboratory Neurorehabilitation Division Neuroscience and Neurorehabilitation Departement Bambino Gesù Children's Hospital, IRCCS Via Torre di Palidoro, 00050 - Passoscuro Fiumicino (Rome) Italy
Data accessibility	With the article
Related research article	S. Summa, G. Tartarisco, M. Favetta, A. Buzachis, A. Romano, G.M. Bernava, G. Vasco, G. Pioggia, M. Petrarca, E. Castelli, E. Bertini, T. Schirinzi, Validation of low-cost system for gait assessment in children with ataxia, Comput Methods Programs Biomed. In Press

## Value of the Data

• To the best of our knowledge, we were the first to use the Kinect in a population of **young ataxic patients** in order to identify novel potential biomarkers for clinical purposes.
• Both clinical and engineer people could be interested in this data because of their rare condition. The prevalence of pediatric ataxias in Europe was estimated in 2013 to be 26/100.000 in children aged 0-19 years.
• These data are useful to have an idea on how much variable are ataxic gait parameters for testing the effect size between subjects. Moreover, these data are suitable for other analysis such as multiple regressions and machine learning models since we included into the dataset not only spatio-temporal features but also scores clinical validated (SARA) for the assessment of severity of Ataxia
• Actually, the therapeutic scenario or other symptomatic interventions lacks. The development of effective therapeutic interventions, tailored to ataxia young patients, and the subsequent clinical trials realization however strictly depends on to the identification of specific and reliable biomarkers.
• Sharing of these data will support replication studies intended to validate and improve our proposed technology.
• There is the need of new technology, using novel methods, that lead to a low cost, easy-accessible system for outpatients and at home usage.

## 1 Data Description

The dataset is reported in a table. Each row is a participant. The first column is the participant number. The second column a categorical variable named Group. Its labels are PA, CA and H that respectively indicates belonging to the group of the progressive, chronic ataxia or healthy participant. We adopted this grouping method to test different classification algorithm, see [1]. The column Diagnosis provide the clinical condition of each patient allowing a different grouping method if needed. In Table 1 the acronyms of each diagnosis are defined.

**Table 1:** Diagnosis acronym legend

Diagnosis	Acronym
Undiagnosed cerebellar atrophy/hypoplasia	UCA
Secondary ataxia due to posterior cranial fossa tumor	Secondary ataxia
Friedreich's ataxia	FRDA
Autosomal recessive spastic ataxia of Charlevoix-Saguenay	ARSACS
Ataxia–telangiectasia syndrome	AT
Spinocerebellar ataxia	SCA
Spinocerebellar ataxia type 2	SCA2
Joubert syndrome	Joubert S.
Others genetic ataxias	PMMA, ADCK3, ITPR1

The column named SARA is the Scale for the Assessment and Rating of Ataxia [2] score provided by a trained clinician during the outpatient visit. Then the remaining columns are the spatio-temporal parameters validated and corrected by the Bland-Altman test in comparison with a standard motion capture system [3]. The definition of the spatio-temporal parameters selected, and a pseudo-code is provided in Table 2.

**Table 2:** List of the spatio-temporal parameters, definitions, and pseudo code to compute them.

Variable name	Variable definition	Formula
Cadence [steps/min]	The rate at which a person walks, expressed in steps per minute.	$\frac{2\;steps*60}{Stride\;Time}$
Speed [m/s]	Mean velocity of progression.	$\frac{Stride\;Length}{Stride\;Time}$
Stride Length [m]	Longitudinal distance from one foot strike to the next one of the same limb.	$ankle_{z}^{FC}-ankle_{z}^{IC}$
Stride Time [s]	Total time that begins with initial and final contact of the same limb.	$\frac{\left(time^{FC}-\;time^{IC}\right)}{Frame\;rate}$
Base Width [m]	Transversal distance between the right and left foot.	$left\;ankle_{x}^{IC}-right\;ankle_{x}^{IC}$ Or$right\;ankle_{x}^{FC}-left\;ankle_{x}^{FC}$
Step Length [m]	Longitudinal distance from one foot strike to the next one.	Considering a left stride:$left\;ankle_{z}^{FC}-right\;ankle_{z}^{IC}$ Or$right\;ankle_{z}^{IC}-left\;ankle_{z}^{IC}$
Stance Phase [% cycle]	Percentage of gait cycle that begins with initial contact and ends at toe-off of the same limb	$\%cycle^{FO}-\;\%cycle^{IC}$
Swing Phase [% cycle]	The period during which the foot is in the air for the purpose of limb advancement	$\%cycle^{FC}-\;\%cycle^{FO}$
Double Support Phase [% cycle]	Time in which both feet are in contact with the floor.	Considering a left stride:$\%cycle_{left}^{FO}-\;\%cycle_{right}^{IC}$ And$\%cycle_{right}^{FO}-\;\%cycle_{left}^{FC}$

## 2 Experimental Design, Materials and Methods

### 2.1 Subjects

The study population was enrolled at the Unit of Neurorehabilitation – Department of Neurosciences of Bambino Gesù Children's Hospital (Rome, Italy), in 2018, and included 51 individuals: 31 patients and 20 healthy subjects (H). A complete description of the patients’ demographic information is reported in [1]. H group included sex/age-matched healthy volunteers without personal/familial history of neurological diseases and no signs at clinical examination (age 14.12(9.1); 12F/8M). Patients were further divided in a PA group (*n*=15) and CA group (*n*=16) depending on diagnosis and clinical course. All patients had genetically confirmed diagnosis and a routine diagnostic work-up, including general and neurological examination, brain MRI, sensory evoked potentials, nerve conduction study and visual acuity evaluation; moreover, they were in follow-up at our center for at least 2 years, in order to ensure a correct group classification. None of the enrolled subjects had relevant cognitive impairment or were taking psychoactive drugs (other usual medications, such as vitamin or antioxidant were allowed). Patients with severe disability, moderate-severe cognitive impairment affecting tests execution, brain and/or cerebellar lesions were excluded. Expert personnel performed clinical assessment. Enrolled patients first received clinical evaluation. Then, all the 51 subjects underwent Kinect-based assessment.

### 2.2 System set-up and protocol

The Kinect v2 was placed on a tripod (tilt angle 0°), in front of the participant so that the frontal view was obtained and at a height of 1 m. It has been suggested that the gait track should be ranged from 1.5 m to 3.5 m from the Kinect [6]. Kinect v2 framerate is 30 frame per second. In order to ensure that a minimum of one full gait cycle per leg was captured we placed the Kinect at a distance of 4.5 m from the starting line and we asked to the patient to walk barefoot at their self-selected speed until the stopping line that was placed 1.5 m from the Kinect, avoiding from the gait cycle analyzed the starting and ending acceleration and deceleration phases.

The 25 anatomical landmarks, including the spine base, left/right hip, left/right knee, and left/right ankle, and the left/right foot (representing the toes), were recorded.

### 2.3 Data analysis

Kinect's markers time series were smoothed with a 4th order Savintzky-Golay filter [4] with a 0.96s time window. Although the cut-off frequency of around 1.5 Hz is low with respect to movement analysis standards, this value deal sufficiently with the low accuracy of the Kinect v2 sensor [5]. While the standard motion capture system uses the force platform to identify the gait events that define a cycle, the Kinect does not offer any automatic data about the timing of interaction between the floor and the feet. Other methods have been proposed to estimate automatically the timing from the limb displacement, the velocity or acceleration [7]. In order to identify gait events, we looked at the evolution of the horizontal displacement differences between hip and foot of the Kinect markers. In particular, we identified the Initial Contact (IC) and the Final Contact (FC) as the maximum and the FootOff (FO) as the minimum. We tested the accuracy of this algorithm measuring the mean absolute error of gait events recognition in three standard gaits with respect the gait events recognized from the system that uses the force platforms. Motion capture system data were computed by Nexus 2.7 (Vicon, Oxford UK) following the c3d format convention.

The list of variables that were selected to detect the main strategies adopted by the patients while walking is described in Table 2. These were computed by a series of routines written in MATLAB environment (Mathworks, Natick MA) for both acquisitions.

### 2.4 Statistical analysis

To test if there was a significant difference between the spatio-temporal parameters obtained by the two measures we looked at correlation and agreement tests. In other words, we looked at the Pearson coefficient (r) hypothesizing a linear relationship between the same parameter but acquired with the two systems. And we used, for each spatio-temporal parameter, the Bland-Altman test to quantify the agreement of the Kinect measures comparing it with the standard motion capture system that is the Vicon system, looking at possible fixed or proportional bias and at the Limits of Agreement (LoA) [8,9]. In case of bias we applied the appropriate corrections. In particular the fixed biases were removed and when a proportional bias was shown the parameter was log-transformed [9]. The results of this validation between the two measures are reported in [1]. The dataset is corrected as just described and is provided in a table by the supplementary materials.

## 3 Ethics Statement

The research conformed to the ethical standards laid down in the 1964 Declaration of Helsinki. All subjects participated on a voluntary basis, after that they or their legal responsible signed the informed consent (the study was approved by local ethical committee).

Fig. 1

**Fig. 1:**
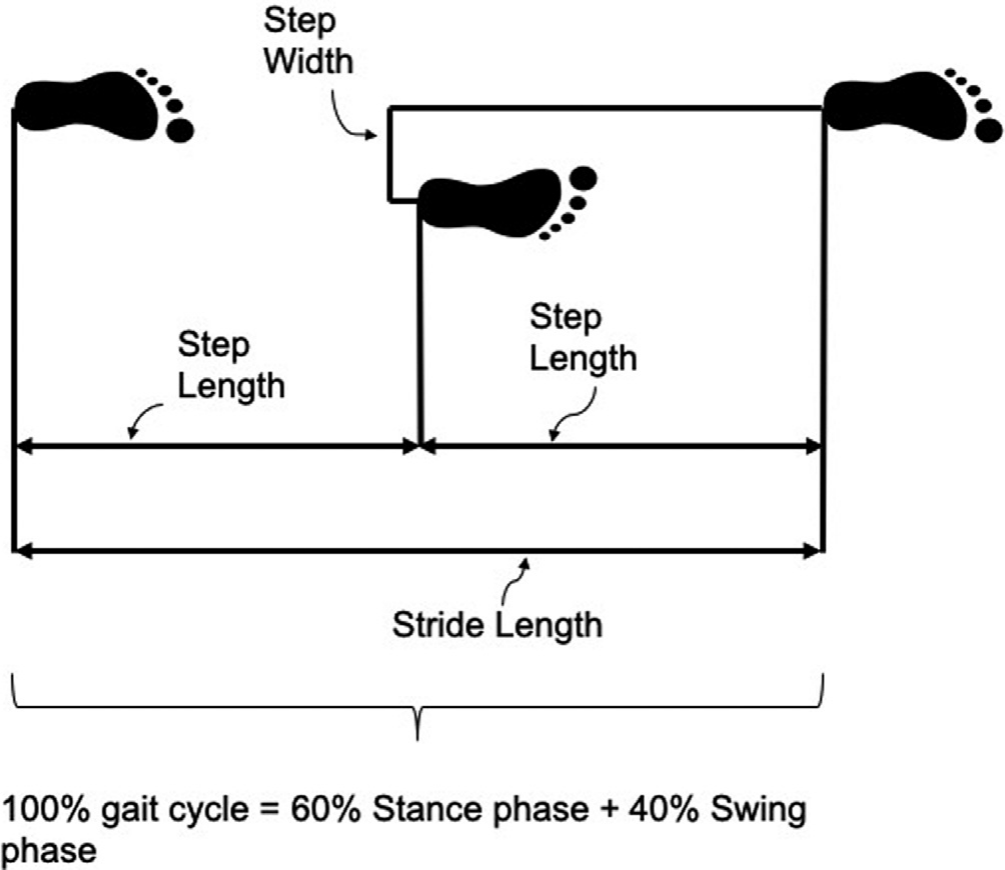
Illustration of the most salient measures of spatio-temporal parameters in the gait cycle.

## Declaration of Competing Interest

The authors declare that they have no known competing financial interests or personal relationships which have, or could be perceived to have, influenced the work reported in this article.

## Appendix Supplementary materials

Image, application 1
